# N-Acetylcysteine Alleviates the Progression of Chronic Kidney Disease: A Three-Year Cohort Study

**DOI:** 10.3390/medicina59111983

**Published:** 2023-11-10

**Authors:** Ai-Hua Chiu, Chih-Jen Wang, Ya-Ling Lin, Chia-Liang Wang, Tsay-I Chiang

**Affiliations:** 1 Department of Nephrology, Kuang-Tien General Hospital, Taichung 433, Taiwan; lovereona0403@gmail.com; 2 Geriatric Medicine Division, Department of Internal Medicine, Changhua Christian Hospital, Changhua City 500, Taiwan; 163532@cch.org.tw; 3 Department of Nursing, Tajen University, Pingtung 907, Taiwan; poiuppoiup@gmail.com; 4 Department of Nutrition, Hungkuang University, Taichung 403, Taiwan; 5 Department of Nursing, Hungkuang University, Taichung 403, Taiwan

**Keywords:** N-acetylcysteine, chronic kidney disease, end-stage renal disease

## Abstract

*Background and Objectives*: The prevalence of chronic kidney disease (CKD) is approximately 10% of the population in many countries. CKD progresses to end-stage renal disease (ESRD), resulting in adverse outcomes, prolonged hospitalization, and increased healthcare costs. Therefore, reducing CKD progression to ESRD is recognized as an important health issue. *Materials and Methods*: Data from the study participants with stage 3 to stage 5 CKD (n = 7668) were collected from the National Health Insurance (NHI) program in Taiwan (1 November 2014 to 31 December 2020). CKD patients who had ingested or not ingested N-acetylcysteine (NAC) for three years were divided into the study group (NAC users; n = 165) and the control group (NAC non-users; n = 165) to explore whether NAC use could alleviate CKD progression and reduce the risks associated with hemodialysis in CKD patients. *Results*: The levels of serum creatinine (SCr) and estimated globular filtration rate (eGFR) were nearly unchanged and/or slightly changed in NAC users, but the SCr levels were slightly increased, and the eGFR levels were significantly decreased in NAC non-users at the six-month interval during the three years. A statistical difference was observed between the two groups for both levels from 12 months to 36 months. The incidence rate of hemodialysis was significantly lower in NAC users than in non-NAC users (4.8% vs. 12.7%, Wald test = 5.947, *p* = 0.015, OR = 34.9). These results indicated that NAC use may improve renal function of CKD patients by modulating SCr and eGFR and, in turn, reducing the risk of hemodialysis. *Conclusions*: We investigated whether NAC could be used to reduce CKD progression to ESRD. For the three-year retrospective study, the incidence rate of hemodialysis was significantly lower in NAC users than in non-NAC users via modulating SCr and eGRF levels. NAC use might be a useful clinical approach for reducing CKD progression to ESRD.

## 1. Introduction

Chronic Kidney Disease (CKD) is a persistent and irreversible kidney damage characterized by abnormalities in kidney structure or function, which endure for more than three months to have significant impacts on individuals’ health [1]. Chronic kidney disease (CKD) is a worldwide public health issue. Epidemiological studies have indicated a CKD prevalence of approximately 13.1% of the population in the United States, 12.9% in Japan, and 10.8% in China [1]. In Taiwan, the national prevalence of CKD is 11.9%, and a marked increase in CKD prevalence is apparent in people older than 60 years. In the annual report of the United States Renal Database System (USRDS) in 2020, Taiwan had the highest ranking for the incidence of dialysis worldwide [2,3]. Therefore, a clinical approach is needed to alleviate the progression of CKD worldwide.

Previous studies have demonstrated that chronic renal failure is associated with oxidative stress [4,5]. An antioxidant-deficient diet increased the progression of renal diseases in animals with nephrectomy [6]. Levels of malondialdehyde (MDA), a product of lipid peroxidation, have been shown to increase after 5/6 nephrectomy in rats [7]. We are therefore conceivable that CKD progression may be mediated through oxidative stress-induced chronic renal failure may associate with oxidative stress. In addition, elevated levels of inflammatory markers, interleukin-6 (IL-6), tumor necrosis factor-alpha (TNF-α), and C-reactive protein (CRP) in patients with chronic kidney disease (CKD) may induce oxidative stress, thereby accelerating the progression of renal damage. However, the detailed mechanism by which oxidative stress affects CKD pathogenesis remains unclear [8,9].

The antioxidant N-acetylcysteine (NAC) is a source of sulfhydryl groups in cells and, due to its interaction with ROS, is a scavenger of free radicals [10]. However, inconsistent effects of NAC on CKD have been reported previously. For example, NAC had no effect on proteinuria, surrogate markers of tubular injury, or renal fibrosis in non-diabetic patients with CKD [11]. In contrast, the administration of NAC 1200 mg twice daily for two weeks resulted in a significant improvement in residual renal function in a small number of hemodialysis (HD) patients (n = 20) [8]. A retrospective study indicated that NAC use was associated with a reduced risk of progression to end-stage renal disease (ESRD) [12]. In the present study, we enrolled 554 patients with CKD stages 3–5 who had used or not used NAC for three years, and we collected data for renal function—serum creatinine (SCr) and the estimated glomerular filtration rate (eGFR) every 6 months for three years to explore whether NAC use could improve renal function and reduce the risks associated with hemodialysis (HD).

## 2. Materials and Methods

### 2.1. Study Participants

The study participants with stage 3 to stage 5 CKD (n = 7668) were collected from the National Health Insurance (NHI) program in Taiwan (1 January 2014 to 31 December 2020). The study participants were older than 20 years, and NAC was taken (NAC users, n = 265) or not taken (NAC non-users, n = 289) for three years. The dosage of NAC was 600 mg orally twice per day for three years. All patients received chronic kidney disease health education during follow-up appointments, which encompassed topics such as nutritional diet, disease-specific medications, and lifestyle guidance.

**Figure 1:**
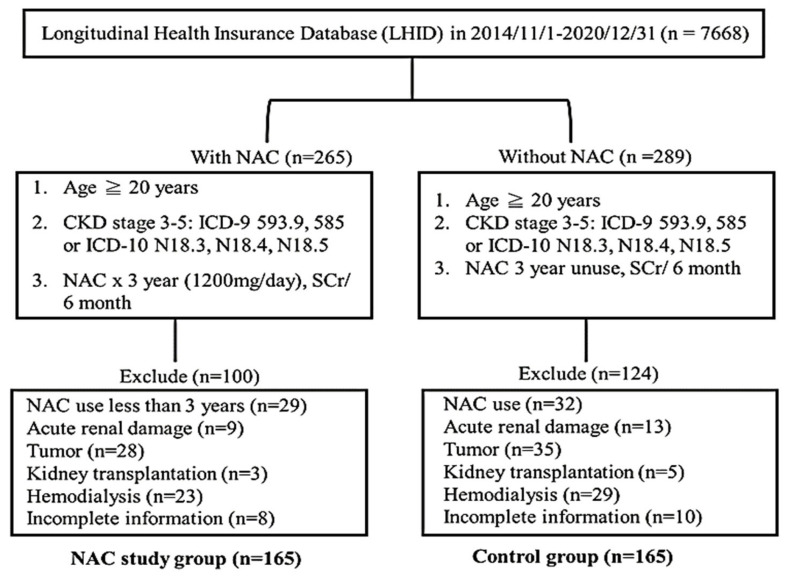
The study flow of the participants in this study.

The levels of SCr and eGFR in all participants were evaluated every six months for three years. Exclusion criteria for NAC users (n = 265) were: (I) NAC was taken before 1 January 2014 (n = 29), (II) acute renal damage (n = 9), (III) malignancy (n = 28), (IV) incomplete cases (n = 8), (V) kidney transplantation (n = 3), and (VI) dialysis before 1 January 2014 (n = 23). Finally, 165 NAC users were enrolled in the study group. Exclusion criteria for NAC non-users (n = 289) were: (I) NAC was taken before 1 January 2014 (n = 32), (II) acute renal damage (n = 13), (III) malignancy (n = 35), (IV) kidney transplantation (n = 5), (V) dialysis before 1 January 2014 (n = 29), and (VI) incomplete cases (n = 10). Therefore, 165 non-users were enrolled into the control group. The study flow diagram for participant selection is shown in Figure 1.

### 2.2. Ethics Statement

The National Health Research Institutes maintain the NHI Research Database (NHIRD), which contains all claims data. The information about the participants included sex, date of birth, medical services received, comorbidities, history of drug use, etc. The study was approved by the Institutional Review Board, Kung Tien General Hospital (KTGH No: 11005).

### 2.3. Determination of SCr and eGFR Levels in Enrolled CKD Patients

We collected venous blood from the enrolled CKD patients to determine the levels of SCr and eGFR at 0, 6, 12, 18, 24, 30, and 36 months during the three years. Blood serum collected from CKD patients to evaluate the levels of SCr. SCr levels were measured using a Beckman Coulter DxC 800 (Kung-Tien General Hospital, Taichung, Taiwan, ROC). eGFR was calculated using serum creatinine and other factors, such as age and gender. The level of eGFR was calculated using the four-variable MDRD formula: eGFR = 186 × [serum creatinine (mg/dL)] − 1.154 × (age) − 0.203 × (0.742 if female).

### 2.4. Statistical Analysis

Statistical analysis was performed using the SPSS statistical software for Windows (Version 22.0). The chi-square test and independent t-sample test were used to assess the difference between the study and control group. *p* < 0.05 was defined as statistically significant.

## 3. Results

### 3.1. Demographic Characteristics and Comorbidities in Patients with CKD with and without NAC Use

Table 1 lists the demographic characteristics and comorbidities in CKD patients with NAC use (n = 165) and without NAC use (n = 165) from 1 November 2014 to 31 December 2020. None of these variables, including age, gender, cancer stage, SCr, eGFR, comorbidities, and history of drug use, differed between CKD patients with and without NAC use. This study design allowed the investigation of whether NAC use could improve renal function in patients with stage 3 to 5 CKD who might progress to ESRD.

### 3.2. Effect of NAC on Renal Function in Patients with CKD

We examined the possibility that NAC could reduce SCr and elevate eGFR levels in patients with CKD at six-month intervals during the three years. The levels of SCr in patients with CKD were slightly increased in NAC users but gradually increased in NAC non-users at each six-month interval during the three years (NAC user: F = 4.701, *p* = 0.0001; NAC non-user: 16.444, *p* = 0.0001; Table 2). In contrast, the levels of eGFR in patients with CKD were gradually decreased in NAC users but markedly decreased in NAC non-users at each six-month interval during the three years (NAC users: F = 3.500, *p* = 0.002; NAC non-users: F = 72.361, *p* = 0.0001; Table 2). Interestingly, different levels of SCr and eGFR were observed between NAC users and NAC non-users from 12 months to 36 months (SCr: *p* = 0.029 for 12 months, *p* = 0.002 for 18 months, *p* = 0.001 for 30 months, *p* = 0.000 for 36 months; eGFR: *p* = 0.006 for 12 months, *p* = 0.025 for 18 months, *p* = 0.001 for 24 months, *p* < 0.0001 for 30 months and 36 months; Table 2). These results suggested that the renal function of patients with CKD might be improved by NAC use over a three-year duration, although kidney function may not be fully recovered.

### 3.3. Effect of NAC on Renal Function in Patients with Different Stages of CKD

We also explored whether NAC could modulate the levels of Scr and eGFR in patients with different stages of CKD. Patients with stage 3a CKD showed gradually increasing SCr levels and gradually decreasing eGFR levels at each six-month interval during the three years (Table 3). The levels of SCr and eGFR differed between NAC users and non-users from 12 to 36 months in patients with stage 3a CKD, and a similar observation was made for the entire study population (Table 2). However, the levels of SCr and eGFR in patients with stage 3b, stage 4, and stage 5 CKD were statistically different between NAC users and non-users from 24 to 36 months (Table 4, Table 5 and Table 6). These results indicated that renal function may be improved by NAC use in patients with different stages of CKD.

### 3.4. Effect of NAC on the Incidence Rate of Hemodialysis in Patients with CKD

We also examined whether NAC consumption could reduce the incidence rate of hemodialysis in patients with CKD. As shown in Table 7, the number of patients with CDK requiring hemodialysis was higher among the non-NAC users than among the NAC users (21 of 165, 12.7% vs. 8 of 165, 4.8%, *p* = 0.015, OR = 34.9). These results indicate that three years of NAC use may provide a 2.6-fold reduction in the incidence rate of hemodialysis in patients with CKD.

### 3.5. Effect of NAC on the Renal Function of diabetic Kidney Disease (DKD) and Non-Diabetic Kidney Disease CKD (Non-DKD)

We enrolled 115 DKD and 50 non-DKD patients to explore whether NAC could reduce the levels of SCr and eGFR and improve renal function in patients with DKD and non-DKD patients. The levels of SCr in patients with DKD were gradually elevated at six-month intervals during the three years (F = 3.537, *p* = 0.002, Table 8), but no similar elevation was observed in the patients with non-DKD. The levels of eGFR were significantly reduced at the six-month interval during the three years in the patients with DKD (F = 2.682, *p* = 0.014, Table 8), but not in the patients with non-DKD. However, statistically significant differences between DKD and non-DKD patients were only revealed at 0 months for SCr (*p* = 0.032), and at 6 months for eGFR (*p* = 0.029). Overall, NAC did not improve renal function in patients with DKD or non-DKD.

## 4. Discussion

Most NAC studies have focused on protection against contrast-induced renal damage, but previous reports show inconsistent findings [13,14,15,16,17]. Few studies have explored whether NAC use could reduce the risk of progression of CKD to ESRD. A meta-analysis was conducted to analyze the efficacy and safety of NAC in the treatment of CKD. The results showed that NAC did reduce cardiovascular events among people with CKD. More interestingly, eGFR and SCr were found to be statistically significantly better in the NAC group compared with the control group [18]. This finding may support our present study, showing that NAC use for three years may improve the renal function of CKD patients by modulating the levels of SCr and eGFR, thereby reducing the risks faced by patients with CKD when undergoing hemodialysis.

Previously, short-term treatment with N-acetylcysteine (NAC), whether administered orally, intravenously, or at high doses, did not improve renal function [19,20,21]. However, a retrospective case-control study indicated that continuous use of NAC at a daily dose of 1200 mg for 90 days reduced the risk of progression of chronic kidney disease [16]. The levels of SCr and eGFR were significantly changed by NAC use from 12 to 36 months (Table 2). The changes in SCr and eGFR levels by NAC use in patients with stage 3a CKD were also observed from 12 months to 36 months (Table 3), and the levels of both SCr and eGFR were changed by NAC use in patients with stage 3b, stage 4, and stage 5 CKD from 24 to 36 months (Table 4, Table 5, Table 6 and Table 7). These results clearly indicate that NAC use may improve renal function in patients with CKD who consume NAC for one or two years. Therefore, NAC use should continue for 2 years for protection of renal function in patients with CKD.

NAC used had no effect on renal function in the present study (Table 8). Over a three-year period, DKD patients using NAC experienced a gradual increase in SCr every six months (F = 3.537, *p* = 0.002) and a significant decrease in eGFR (F = 2.682, *p* = 0.014). In contrast, NAC use had no significant impact on SCr and eGFR in non-DKD patients. When comparing NAC users and non-users, DKD patients showed significant differences in SCr and eGFR at the 24th, 30th, and 36th months. Notably, NAC use in DKD patients resulted in a significant increase in SCr every six months (F = 3.537, *p* = 0.002) and a significant decrease in eGFR (F = 2.682, *p* = 0.014). However, NAC had no significant effect on renal function in non-DKD patients. Overall, while NAC did not improve renal function in either DKD or non-DKD patients, it did not lead to a significant deterioration in non-DKD patients. A previous retrospective study indicated that NAC use was associated with a reduced risk of progression to ESRD, but this was shown in non-DKD, not in DKD patients [12]. This conflicting finding could reflect the small number of non-DKD patients (n = 50) enrolled in the present study compared to the previous report (n = 1354) [16].

NAC use might be a beneficial clinical approach to prevent the progression of CKD to ESRD. However, this study was subject to potential uncontrollable biases as it relied on clinical data from a retrospective analysis of cases over a three-year period, following N-acetylcysteine (NAC) drug protocol conformity in patients with CKD and concurrent pulmonary diseases. Furthermore, the cases lacked comprehensive proteinuria data, which could not be included in the analysis and therefore remains an unaccounted-for factor. Therefore, it is necessary to plan prospective, randomized, double-blind, placebo-controlled trials and longer-term follow-up studies in the future to confirm the observations of the present study.

## 5. Conclusions

NAC use for two to three years may reduce the risk of progression of CKD to ESRD by modulating the levels of SCr and eGFR. NAC may potentially reduce the risk of CKD progression to ESRD in patients with non-DKD. Nevertheless, NAC use might be a beneficial and safe clinical approach to prevent the progression of CKD to ESRD. We, therefore suggest that early intervention with NAC may be helpful for reducing CKD progression to ESRD.

## Figures and Tables

**Table 1:** Demographic characteristics and comorbidities in CKD cohorts with or without NAC use.

Variables	NAC Use		*p* Value
	Yes (n = 165)	No (n = 165)	
Age (year)	73.59 ± 12.26	72.58 ± 10.03	0.415
Gender			
Male	78 (47.3)	77 (46.7)	0.012
Female	87 (52.7)	88 (53.3)	
CKD stage			
3a	65 (39.4)	67 (40.6)	0.135
3b	56 (33.9)	56 (33.9)	
4	38 (23.0)	37 (22.4)	
5	6 (3.6)	5 (3.0)	
Renal function			
SCr	1.83 ± 0.78	1.79 ± 0.79	0.629
eGFR	38.32 ± 12.89	39.52 ± 13.07	0.404
Comorbidity			
DM	115 (69.7)	113 (68.5)	0.812
Hypertension	146 (88.5)	137 (83.0)	0.156
Stroke	48 (29.1)	52 (31.5)	0.632
CVD	118 (71.5)	115 (69.7)	0.717
Drug history			
ACE1 (ARB)	127 (77.0)	126 (76.4)	0.896
SGLT2i	9 (5.5)	5 (3.0)	0.275
Pentoxifylline	84 (50.9)	96 (58.2)	0.371
HCO_3_^−^	98 (59.4)	106 (64.2)	0.314
Vamin D	48 (29.1)	59 (35.8)	0.207
Kremezin	78 (47.3)	66 (40.0)	0.317

**Table 2:** Effect of NAC on the renal function of patients with CKD.

Variables	NAC Users (n = 165)	NAC Non-Users (n = 165)	*p* Value
SCr			
0 M	1.83 ± 0.78	1.80 ± 0.79	0.629
6 M	1.82 ± 0.81	2.12 ± 2.48	0.096
12 M	1.81 ± 0.81	2.25 ± 2.72	0.029
18 M	2.03 ± 1.66	2.17 ± 1.09	0.19
24 M	2.00 ± 1.11	2.52 ± 1.79	0.002
30 M	2.05 ± 1.43	2.70 ± 1.95	0.001
36 M	2.13 ± 1.54	3.33 ± 2.49	0.0001
F/*p*	4.701/0.0001	16.444/0.0001	
eGFR			
0 M	38.32 ± 12.89	39.39 ± 13.09	0.404
6 M	38.61 ± 12.87	36.80 ± 12.30	0.24
12 M	38.98 ± 12.99	34.96 ± 13.34	0.006
18 M	37.32 ± 13.42	33.95 ± 13.70	0.025
24 M	36.31 ± 13.20	31.44 ± 13.90	0.001
30 M	37.10 ± 14.00	30.38 ± 14.66	0.0001
36 M	36.71 ± 14.58	26.70 ± 15.63	0.0001
F/*p*	3.500/0.002	72.361/0.0001	

M; month.

**Table 3:** Effect of NAC on renal function of patients with stage 3a CKD.

Variables	NAC Users (n = 65)	NAC Non-Users (n = 67)	*p* Value
SCr			
0 M	1.30 ± 0.26	1.27 ± 0.20	0.444
6 M	1.40 ± 0.32	1.42 ± 0.30	0.751
12 M	1.38 ± 0.28	1.54 ± 0.54	0.029
18 M	1.51 ± 0.40	1.69 ± 0.88	0.143
24 M	1.56 ± 0.39	1.93 ± 1.47	0.053
30 M	1.58 ± 0.63	2.01 ± 1.49	0.034
36 M	1.58 ± 0.75	2.56 ± 2.21	0.001
F/*p*	6.596/0.0001	15.402/0.0001	
eGFR			
0 M	51.06 ± 3.95	51.97 ± 4.33	0.21
6 M	47.81 ± 8.25	46.57 ± 7.25	0.358
12 M	48.68 ± 7.75	43.98 ± 9.42	0.002
18 M	45.18 ± 9.92	42.17 ± 10.96	0.103
24 M	43.87 ± 9.32	39.66 ± 11.65	0.025
30 M	44.32 ± 10.23	38.12 ± 12.08	0.002
36 M	45.18 ± 10.86	35.51 ± 15.05	0.0001
F/*p*	9.122/0.0001	34.554/0.0001	

M; month.

**Table 4:** Effect of NAC on renal function of patients with stage 3b CKD.

Variables	NAC Users (n = 56)	Non-Users (n = 56)	*p* Value
SCr			
0 M	1.70 ± 0.46	1.66 ± 0.28	0.596
6 M	1.68 ± 0.49	2.31 ± 3.98	0.244
12 M	1.71 ± 0.55	1.93 ± 0.68	0.062
18 M	2.00 ± 2.37	1.92 ± 0.68	0.822
24 M	1.77 ± 0.62	2.16 ± 1.39	0.058
30 M	1.77 ± 0.71	2.31 ± 1.79	0.038
36 M	1.84 ± 0.75	2.89 ± 2.29	0.002
F/*p*	0.762/0.601	2.910/0.009	
eGFR			
0 M	36.91 ± 5.23	38.21 ± 3.91	0.14
6 M	38.62 ± 9.12	37.18 ± 8.40	0.388
12 M	38.25 ± 9.70	35.45 ± 9.94	0.134
18 M	38.64 ± 10.31	35.63 ± 9.91	0.119
24 M	37.06 ± 10.44	32.81 ± 10.35	0.033
30 M	38.04 ± 11.77	32.87 ± 12.31	0.025
36 M	37.11 ± 11.96	26.92 ± 12.71	0.0001
F/*p*	0.752/0.608	4.743/0.003	

M; month.

**Table 5:** Effect of NAC on renal function of patients with stage 4 CKD.

Variable	NAC Users (n = 38)	NAC Non-Users (n = 37)	*p* Value
SCr			
0 M	2.59 ± 0.53	2.59 ± 0.64	0.999
6 M	2.62 ± 0.83	3.00 ± 2.20	0.329
12 M	2.53 ± 0.85	4.18 ± 5.98	0.106
18 M	2.74 ± 1.06	3.53 ± 3.19	0.154
24 M	2.74 ± 1.12	4.28 ± 3.83	0.024
30 M	2.95 ± 1.74	4.63 ± 3.76	0.015
36 M	3.17 ± 1.87	5.15 ± 2.53	0.0001
F/*p*	2.944/0.009	5.709/0.0001	
eGFR			
0 M	22.43 ± 4.66	22.76 ± 5.50	0.782
6 M	23.83 ± 8.25	22.87 ± 7.40	0.598
12 M	24.93 ± 9.05	21.50 ± 8.82	0.101
18 M	23.69 ± 10.38	20.25 ± 8.84	0.127
24 M	24.04 ± 10.90	17.97 ± 9.10	0.011
30 M	24.03 ± 11.83	16.02 ± 8.70	0.001
36 M	22.13 ± 10.31	13.42 ± 8.83	0.0001
F/*p*	1.149/0.335	31.413/0.0001	

M; month.

**Table 6:** Effect of NAC on renal function of patients with stage 5 CKD.

Variable	NAC Users (n = 6)	NAC Non-Users (n = 5)	*p* Value
SCr			
0 M	4.01 ± 0.98	4.43 ± 0.80	0.999
6 M	2.68 ± 2.02	5.10 ± 0.87	0.329
12 M	2.83 ± 2.06	4.35 ± 0.66	0.106
18 M	3.55 ± 2.86	5.06 ± 0.43	0.154
24 M	4.31 ± 3.31	5.45 ± 0.90	0.024
30 M	4.08 ± 4.34	5.93 ± 0.90	0.015
36 M	4.18 ± 4.52	6.13 ± 0.91	0.0001
F/*p*	0.901/0.009	5.178/0.002	
eGFR			
0 M	14.14 ± 6.16	11.30 ±1.53	0.345
6 M	32.48 ± 18.41	9.69 ± 2.01	0.029
12 M	29.70 ± 17.16	11.40 ± 1.07	0.047
18 M	26.13 ± 17.27	9.52 ± 1 10	0.065
24 M	25.07 ± 21.80	8.88 ± 1.57	0.125
30 M	32.85 ± 22.93	8.01 ± 1.27	0.045
36 M	33.67 ± 23.36	7.85 ± 1.49	0.042
F/*p*	2.202/0.076	74.743/0.003	

M; month.

**Table 7:** Demographic characteristics and comorbidities in CKD cohorts with or without NAC use.

Groups	Total	Hemodialysis (%)		Odds (%)
		No	Yes	
NAC users	165	157	8 (4.8)	5.1
NAC non-users	165	144	21 (12.7)	14.6

Wald = 5.947; *p* = 0.015; OR: 34.9.

**Table 8:** Effect of NAC on the renal function of diabetic patients with CKD (DKD) compared to non-diabetic patients with CDK (non-DKD).

Variable	DKD (n = 115)	Non-DKD (n = 50)	*p* Value
SCr			
0 M	1.73 ± 0.65	2.07 ± 0.99	0.032
6 M	1.77 ± 0.74	1.96 ± 0.95	0.202
12 M	1.77 ± 0.76	1.90 ± 0.92	0.357
18 M	2.03 ± 1.86	2.06 ± 1.07	0.917
24 M	1.93 ± 1.05	2.18 ± 1.23	0.171
30 M	2.00 ± 1.34	2.18 ± 1.61	0.468
36 M	2.08 ± 1.49	2.24 ± 1.65	0.559
F/*p*	3.537/0.002	1.757/0.108	
eGFR			
0 M	39.58 ± 12.26	35.44 ± 13.93	0.058
6 M	39.18 ± 12.17	37.31 ± 14.41	0.029
12 M	39.18 ± 12.46	38.51 ± 14.26	0.772
18 M	37.65 ± 12.77	36.56 ± 14.92	0.632
24 M	36.99 ± 12.51	34.75 ± 14.66	0.318
30 M	37.62 ± 13.74	35.90 ± 14.65	0.468
36 M	37.23 ± 14.53	35.53 ± 15.79	0.493
F/*p*	2.682/0.014	1.690/0.123	

M; month.

## Data Availability

The data presented in this study are available in the article.
